# Comparison of GARP and MaxEnt in Modeling Current and Future Geographic Distribution of *Ceracris nigricornis* Walker (Acrididae, Orthoptera) in China

**DOI:** 10.1002/ece3.70439

**Published:** 2024-10-22

**Authors:** Chun Fu, Xuanye Wen, Tingting Huang, Yanli Wang, Xiu Liu, Na Jiang, Rulin Wang, Jinpeng Zhao

**Affiliations:** ^1^ Key Laboratory of Sichuan Province for Bamboo Pests Control and Resource Development Leshan Normal University Leshan People's Republic of China; ^2^ Center for Biological Disaster Prevention and Control, National Forestry and Grassland Administration Shenyang People's Republic of China; ^3^ Chengdu Agricultural Technology Extension Station Chengdu People's Republic of China; ^4^ Sichuan Provincial Rural Economic Information Center Chengdu People's Republic of China; ^5^ Fushun Meteorological Bureau Zigong People's Republic of China; ^6^ College of Tourism and Geographical Science Leshan Normal University Leshan People's Republic of China

**Keywords:** bamboo pest, climate change, niche model, suitable area

## Abstract

*Ceracris nigricornis* Walker is an insect of the Acrididae, which can harm bamboo, rice, corn, sorghum and other crops, and can cause serious economic losses. In this study, based on 234 occurrence sites of *C. nigricornis* obtained from the Global Biodiversity Information Facility and literature, and data of three future climate scenarios presented by CMIP6, two niche models (GARP, MaxEnt) were used to predict the suitable area of *C. nigricornis* in China. The result shows that the main environmental factors affecting the distribution of *C. nigricornis* are min temperature of coldest month (bio6), mean temperature of coldest quarter (bio11), precipitation of driest month (bio14) and precipitation of wettest quarter (bio16). From the result of MaxEnt model, it can be seen that the suitable area of *C. nigricornis* in China is 128.91 × 10^4^ km^2^ under current scenario. It will decrease by 3.19% in the 2050s and then increase by 12.04% in the 2090s under the SSP1‐2.6 scenario, increase by 5.79% in the 2050s and then decrease by 7.53% in the 2090s under the SSP2‐4.5 scenario, and increase by 33.03% in 2050s and then decrease by 23.31% in the 2090s under SSP5‐8.5 scenario. From the result of GARP model, it can be seen that the suitable area of *C. nigricornis* in China is 166.09 × 10^4^ km^2^ under current scenario. It will increase by 8.41% in 2050s and then continue to increase by 6.11% in 2090s under SSP1‐2.6 scenario, increase by 23.84% in the 2050s and then decrease by 0.88% in the 2090s under the SSP2‐4.5 scenario, and increase by 34.37% in 2050s and then decrease by 1.75% in 2090s under SSP5‐8.5 scenario. The boundaries of suitable areas will expand to the north and southwest of China under future climate change scenarios, specially in Sichuan, Chongqing and Yunnan. Local forestry authorities should strengthen the monitoring of bamboo forests to prevent the damage caused by the introduction of *C. nigricornis*.

## Graphical abstract

*Ceracris nigricornis* Walker is a species of grasshopper of the family Acrididae that is a severe pest, which can harm bamboo, rice, corn, sorghum and other crops, and can cause serious economic losses. In this study, based on 234 occurrence sites of *C. nigricornis* obtained from the Global Biodiversity Information Facility and literature, and data of three future climate scenarios presented by CMIP6, two niche models (GARP, MaxEnt) were used to predict the suitable area of *C. nigricornis* in China. The result shows that the main environmental factors affecting the distribution of *C. nigricornis* are min temperature of coldest month (bio6), mean temperature of coldest quarter (bio11), precipitation of driest month (bio14) and precipitation of wettest quarter (bio16). In the future scenarios,The boundaries of suitable areas will expand to the north and southwest of China under future climate change scenarios, specially in Sichuan, Chongqing and Yunnan.

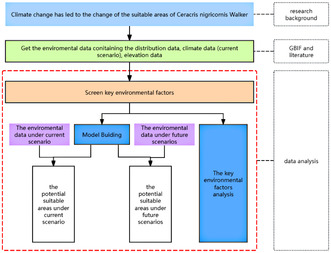

## 1 Introduction

*Ceracris nigricornis* Walker (Orthopteridae: Acrididae) is a severe grasshopper pest of bamboos and is mainly distributed in China, Korea, India, Myanmar, Laos, Vietnam, Nepal and Thailand. There is no literature on whether *C. nigricornis* is a native or exotic species, but some studies suggest that its transmission route in China may start in Yunnan and spread through Guangxi, Hunan, Sichuan, Hubei and Anhui to Shaanxi (Wang 2019). *C. nigricornis* can harm bamboo, rice, corn, sorghum and other crops and can cause serious economic losses (Yuan et al. 2019). The adult and nymphs of *C. nigricornis* feed on the leaves of plant, biting the bamboo leaves into pure toothed‐like grooves and even eating them up. As it can be seen that *C. nigricornis* have become very common in southern China and are very harmful to crops, it is very necessary to study them. Many scholars have studied its biological characteristics (Feng et al. 2024; Gao 2017), but no reports of its disaster risk assessment and regionalization in China have been found, which is an important prerequisite and basis for effectively controlling it.

Niche model, contains two types: time‐based and space‐based, is a mathematical model to study the relationship between various organisms in an ecosystem and their adaptability to the environment. The time‐based niche model generally takes the dynamic change of biological population as the research object, whereas the space‐based niche model mainly studies the space occupation between biological communities. The space‐based niche model include Bioclim (Busby 1991), Domain (Carpenter et al. 1993), GARP (Stockwell and Peters 1999), ENFA (Hirzel et al. 2002), and MaxEnt (Phillips, Anderson, and Schapire 2006; Phillips and Dudik 2008), which the most commonly used are MaxEnt and GARP. MaxEnt model uses the distribution points of species and environmental variables to calculate the ecological niche of species and simulate the potential distribution of species (Xu, Peng, and Peng 2015). It has high prediction accuracy and ease of use (Merow, Smith, and Silander 2013), and has been widely used in the prediction of species potential distribution, the risk assessment of alien species invasion, and the study of the impact of climate change on biodiversity. Combining the optimization ability of genetic algorithm with the environmental interpretation ability of niche model, GARP model provides a powerful tool for predicting species distribution. It can be adapted to various data types and research problems and has been widely used to predict the species distribution of different taxa and pathogens in landscapes since the late 1990s (Haase et al. 2021). Based on the above advantages, MaxEnt model and GARP model as the tools of this study can better predict the potential distribution of *C. nigricornis*.

As a big agricultural country, China is very sensitive to climate change. Climate change not only directly affects the ecological habits of locusts but also indirectly affects the distribution and outbreak of locusts through other environmental factors (Liu, Zhang, and He 2024), which will seriously affect agricultural production and food security. The Climate Model Intercomparison Project (CMIP) is an international collaborative project initiated by the World Climate Research Programme (WCRP), whose aim is to understand past, present and future climate change by collecting and comparing simulation results from various global climate models (GCMs) (Zhou, Zou, and Chen 2019). At present, the 33 research teams involved in CMIP6 have registered 112 versions of the global climate system model. For the southwestern China, the ACCESS−CM2, CMCC−CM2−SR5 and CMCC−ESM2 global climate mode have the best performance in the comprehensive simulation ability of temperature (Jin, Jiang, and Zhang 2022).

In this paper, the Jackknife test method was used to analyze the key environmental factors affecting the survival of *C. nigricornis* and two niche model (MaxEnt and GARP) combined with ArcGIS were used to simulate the potential suitable areas of *C. nigricornis* in China. The purpose of this study is to answer the following questions: What are the key environmental factors affecting the distribution of *C. nigricornis*? What is the relationship between key environmental variables and the probability of the presence of *C. nigricornis*? What are the potential suitable areas of the *C. nigricornis* in China under current climate scenario? and How will climate change in future affecting the potential suitable areas of the *C. nigricornis*? The answers to the above questions is of great significance to research formulate feasible countermeasures to prevent and control *C. nigricornis*, reduce the serious economic loss caused by it, and ensure the stable development of bamboo and crop industry in China.

## 2 Materials and Methods

### 2.1 Data Sources and Processing

**FIGURE 1:**
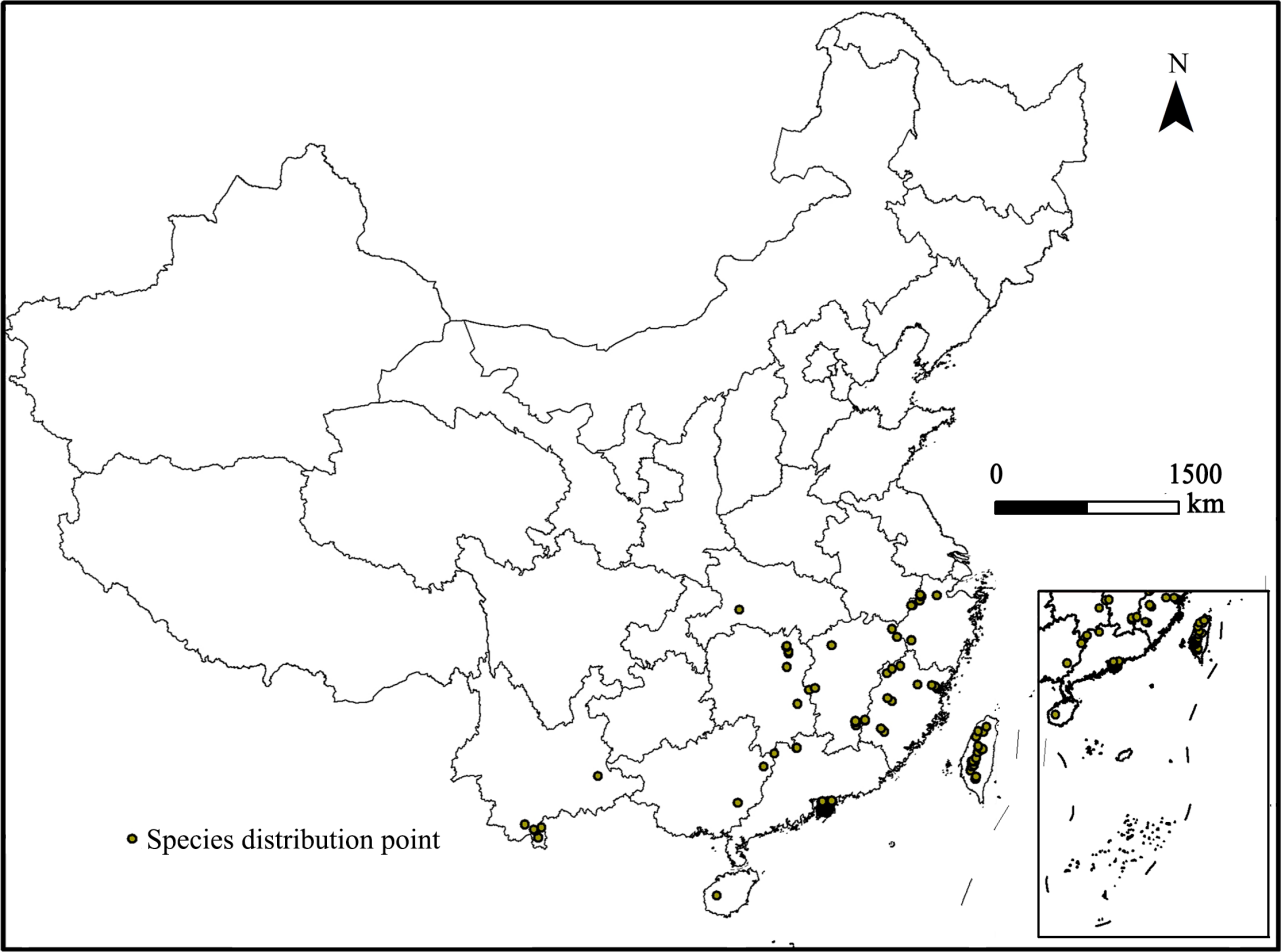
Geographical distribution records of *Ceracris nigricornis* in China.

The distribution records of *C. nigricornis* were derived from the Global Biodiversity Information Facility (GBIF 2023) and literature (Gao, Jiang, and Fan 2020; Wu 2005; He 2002). Google Earth was used to retrieve the records missing the latitude and longitude, and ENMTOOL software was used to remove duplicate records in 2.5 arc‐min grid (Zhu, Liu, and Gao 2014; Sillero and Barbosa 2021). After the abovementioned treatment, 234 occurrence records were reserved for species distribution prediction (Table S1). The specific points are in Figure 1.

In this study, we chose the ACCESS−CM2 model developed by commonwealth scientific and industrial research organization (CSIRO) for future climate simulations. We chose SSP1‐2.6 scenario, SSP2‐4.5 scenario and SSP5‐8.5 scenario to represent different future climate change, of which the SSP1‐2.6 scenario represents global carbon dioxide emissions will significantly reduce in the future, reaching net zero emissions by 2050. The SSP2‐4.5 scenario represents global carbon dioxide emissions will hover around current levels before starting to decline by mid‐century, reaching net zero by 2100. The SSP5‐8.5 scenario represents global carbon dioxide emissions will rise steadily in the future, reaching about double current levels by 2100.

The environmental factors data contain 19 bioclimatic factors and altitude, which can be downloaded from WorldClim (https://www.worldclim.org/). The file format of the environmental factor is tif with a spatial resolution of 2.5 arc‐min grids, and ArcGIS 10.0 was used to convert it to “ASC” format for future use. Contribution rate of environmental factors to predictive model construction was tested by the jackknife method, and the factors with zero contribution rate were deleted. To reduce the impact of multiple contributions of environmental factors on the model and avoid errors in modeling caused by multicollinearity, SPSS 14.0 software was used to conduct Pearson test, and the one with the lower contribution percentage value in the jackknife test among the two factors with correlation coefficients > 0.85 was eliminated. Finally, the variables and their factors used for species modeling are shown in Table 1.

**TABLE 1:** Environmental variables and their factors used for modeling. Climatic factor (bio) and topographic factor (alt).

Code	Environmental factor	Unit
Bio3	Isothermality	—
Bio5	Max temperature of warmest month	°C
Bio6	Min temperature of coldest month	°C
Bio11	Mean temperature of coldest quarter	°C
Bio14	Precipitation of driest month	mm
Bio16	Precipitation of wettest quarter	mm
Alt	Elevation	m

### 2.2 Niche Models and Parameter Settings

The software containing the MaxEnt model was downloaded from the official website (V3.4.4, https://biodiversityinformatics.amnh.org/open_source/maxent/). The geographical distribution records of *C. nigricornis* in “CSV” format was imported into the “Samples” data boxes of MaxEnt software, whereas the environmental factors in “ASC” format was imported into the “environmental layers” data boxes. The options on the right side of the software, such as “Create response curves” and “Do jackknife to measure variable importance”, were selected. The feature combinations (FCs) option and regularization multiplier (RM) option were used to optimize the accuracy of the MaxEnt model. The FCs option contains linear (L), quadratic (Q), hinge (H), product (P) and threshold (T), which can be formed as L, LQ, H, LQH, LQHP and LQHPT. The RM step was set to 0.5 and ranges from 0.5 to 4. Therefore, 48 different combinations of RM and FCs were established and the combinational model with the minimum Akaike information criterion correction (AICc) value is determined to be the optimal model (Guo et al. 2022). “Random test percentage” was set to 25% in the initial model, whereas the “replicates” was set to 10 in the reconstructed model. The software containing the GARP model was also downloaded from the official website (V1.1.6, http://www.lifemapper.org). The training data and the test data set were 75% and 25% of the distribution data, and the number of runs was set to 20. Beyond that, all other basic settings are set to default values.

### 2.3 Model Accuracy Evaluation Method

The ROC curve (Receiver Operating Characteristic curve) is a graphical tool used to evaluate the performance of binary classification models, which demonstrates the performance of the model by plotting True Positive Rate (TPR) and False Positive Rate (FPR) under different classification thresholds. The closer the ROC curve is to the upper left corner, the better the performance of the model, that is, the lower the false positive rate and the higher the correct recognition rate. The area under ROC curve (AUC), the area formed by the ROC curve and the axis, is another important indicator to measure the model performance. Many times the ROC curve does not clearly indicate which classifier performs better, but AUC as a value can perform better. Therefore, we use the AUC value as a criterion for evaluating the model for its discrimination capacity (Lobo, Jiménez‐Valverde, and Real 2008). The closer the value of AUC is to 1, the better the model performance (Xu 2024). If the AUC value is > 0.9, the prediction effect of the model is better; If the AUC value is < 0.9 and > 0.7, the prediction effect of the model is general; If the AUC value is < 0.7, the prediction effect of the model is poor (Merow, Smith, and Silander 2013; Wang et al. 2007; Wang, Zhu, and Xu 2021).

### 2.4 Suitable Area Division

The calculation results in Ascii format of MaxEnt and GARP were loaded in ArcGIS 10.2, and the suitable biological grade was divided and visualized to obtain the potential distribution map of species (The projection coordinate system was GCS_WGS_1984). It is important to select the appropriate threshold value when converting the results of successive species suitability predictions into the boolean form of “suitable area” and “unsuitable area”. A large number of studies have proved that the threshold method of sensitivity‐specificity and maximization approach is significantly superior to other threshold methods (Jiménez‐Valverde and Lobo 2007), and the threshold of the existence probability of a specificity is 0.33. The region with the occurrence probability of species < 0.05 was defined as the unsuitable area of *C. nigricornis*, the region with the occurrence probability of 0.05–0.33 was defined as the lowly‐suitable area, the region with the occurrence probability of 0.33–0.66 was defined as the moderately‐suitable area, and the region with the occurrence probability of 0.66–1.00 was defined as the highly‐suitable area (Sun, Qin, and Liu 2012). The changes of the potential distribution area of *C. nigricornis* were mainly calculated using the SDM toolbox (Etherington 2011), which was mainly written based on python. We calculated the percentage change of suitable area from current to the 2050s and from the 2050s to the 2090s.

## 3 Results

### 3.1 Model Accuracy Evaluation

According to the known occurrence data and current climate data, the potential geographical distribution map of *C. nigricornis* was modeled and the region climatically suitable was determined. The AUC values of two niche models (MaxEnt and GARP) for 10 replicates were all > 0.92 (Table S2), indicating that the performance of the distribution model was better than that of the random model, and the stability between each repetition was good. The model had excellent performance in predicting the suitable area of *C. nigricornis*.

### 3.2 Key Environmental Variables Affecting the Geographical Pattern

**FIGURE 2:**
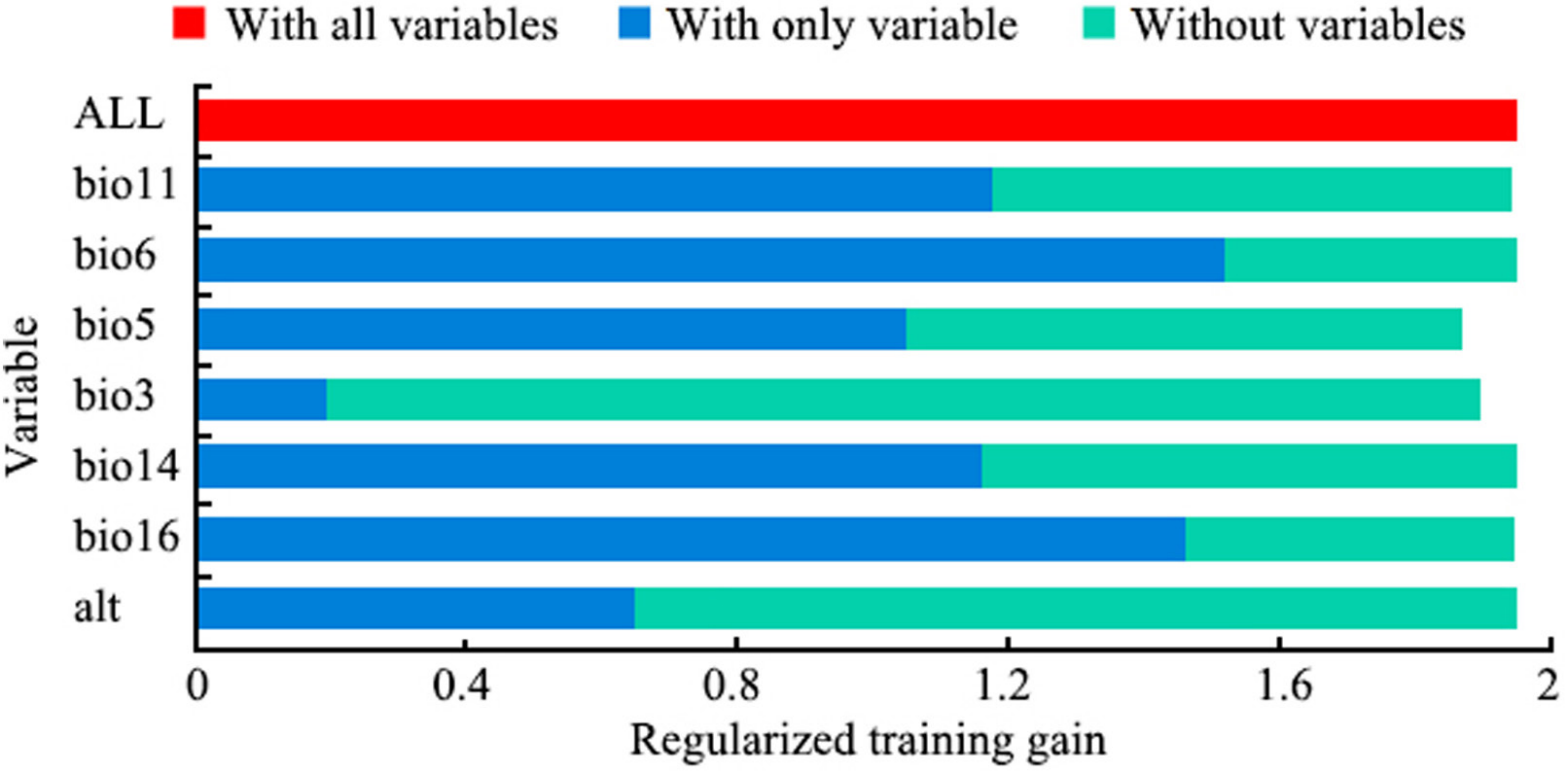
Jackknife test for evaluating the importance of environmental variables by Maxent.

The jackknife method of Maxent software was used to test the importance of seven environment variables to modeling (Figure 2 and Table 2). The results show that the regularization training gain of min temperature of coldest month (Bio6) is 1.55, the percent contribution is 18.3%, and the permutation importance is 39.7%, all of which are high levels. It can be concluded that min temperature of coldest month is the most important variable affecting the distribution of *C. nigricornis*. The regularization training gain value of precipitation of wettest quarter (bio16) is 1.42, and the contribution rate is 44.5%, indicating that this variable is also very important. Similarly, mean temperature of coldest quarter (bio11) and precipitation of driest month (Bio14) are also important factors affecting the distribution of *C. nigricornis*. In the process of variable elimination modeling, the regularized training gain value of isothermality (bio3) and max temperature of warmest month (bio5) decreased the most, indicating that this variable contains important information with the *C. nigricornis* distribution. According to the above comparative analysis, isothermality (bio3), max temperature of warmest month (bio5), min temperature of coldest month (bio6), mean temperature of coldest quarter (bio11), precipitation of driest month (bio14) and precipitation of wettest quarter (bio16), and elevation (alt) are the key environmental factors that affect the distribution of *C. nigricornis*.

**TABLE 2:** The percent contribution and permutation important of environmental variables by Maxent.

Code	Percent contribution (%)	Permutation importance (%)
Bio3	2.1	14.9
Bio5	4.2	20.4
Bio6	18.3	39.7
Bio11	10.4	5.3
Bio14	11.2	4.1
Bio16	44.5	10.5
Alt	9.3	5.1

**FIGURE 3:**
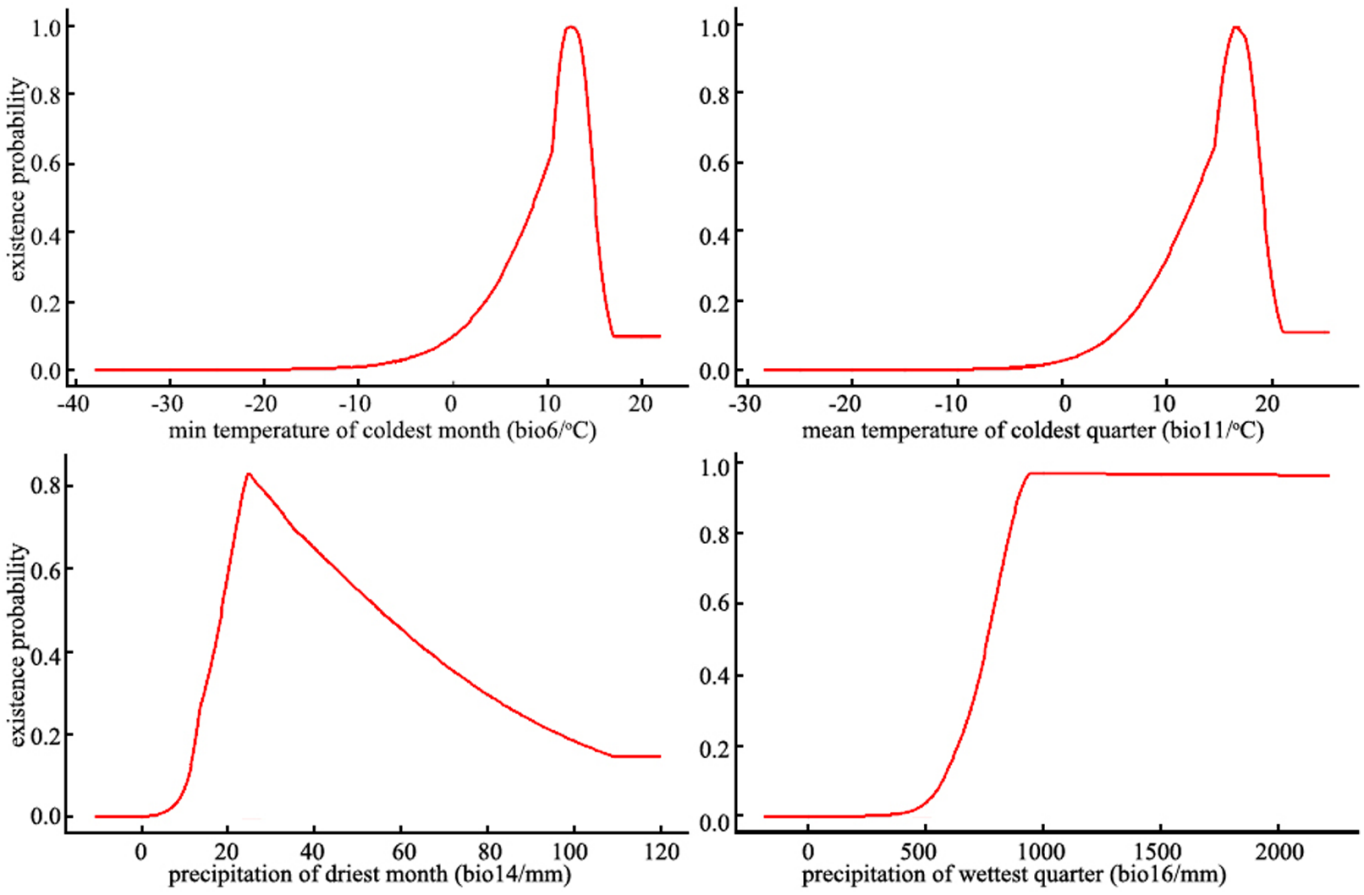
Relationship between the key environmental variables and existence probability of *Ceracris nigricornis* by Maxent.

The key environmental variables affecting the distribution of *C. nigricornis* are precipitation and temperature (Figure 3). In terms of precipitation, when precipitation of driest month (bio14) is < 15 mm or more than 74.3 mm, the existence probability of *C. nigricornis* is < 0.33, and the optimum value is 24.5 mm. According to the classification method of suitable areas, precipitation of driest month (bio14) in the area above the moderately‐suitable area is more than 710.28 mm. In terms of temperature, the optimum min temperature of coldest month (bio6) is 12.5°C, which is lower or higher than this value will reduce the existence probability of *C. nigricornis*. When the value is 6.1°C–15.6°C, the existence probability of *C. nigricornis* is > 0.33. Similarly, when the existence probability of *C. nigricornis* is > 0.33, mean temperature of coldest quarter (bio11) is 10.3°C–19.7°C, and the optimum value is 16.6°C.

### 3.3 Potential Suitable Under Current Scenario

**FIGURE 4:**
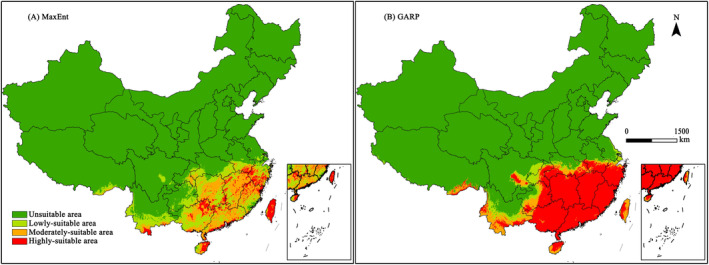
Potential distribution of *Ceracris nigricornis* in China under current scenario according to Maxent (left) and GARP (right).

According to the potential geographical distribution pattern (Figure 4) under current scenario, it can be seen that southeastern China and part of southwestern China were mainly suitable areas for *C. nigricornis*. Compare the prediction result of the two models, the suitable areas all contain Jiangxi, Hunan, Zhejiang and Anhui in the lower‐middle reaches of the Yangtze River, southern Yunnan, Taiwan, Hainan, Guangdong and Guangxi.

From the result of MaxEnt model (Figure 4A), it can be seen that the suitable habitat area of *C. nigricornis* in China is 128.91 × 10^4^ km^2^, accounting for 13.40% of the total land. The highly‐suitable areas are scattered in several provinces in southern Yangtze River, whereas the moderately‐suitable areas are divergent distribution with taking as the center of Poyang Lake plain and Lianghu plain in Xiang‐gan area, including the southeastern Chongqing, the central of Hunan, the eastern of Jiangxi, the southern of Anhui, the western of Zhejiang, the northern of Fujian, the most regions of Guangdong, the northwest of Guangxi and the southern of Yunnan. The area of the highly‐suitable areas, the moderately‐suitable areas and the lowly‐suitable areas account for 2.05%, 5.29% and 6.08% of the total land, respectively. From the result of GARP model (Figure 4B), it can be seen that the suitable habitat area of *C. nigricornis* in China is 166.09 × 10^4^ km^2^, accounting for 17.27% of the total land. The highly‐suitable areas are mainly distributed in Guangdong‐Guangxi region to the Eo‐Wan region, which occupies most of the area of several provinces south of the Yangtze River. The moderately‐suitable areas are mainly distributed in the southern of Yunnan and Guizhou. The area of the highly‐suitable areas, the moderately‐suitable areas and the lowly‐suitable areas account for 12.25%, 3.0% and 2.06% of the total land, respectively.

### 3.4 Potential Suitable Under Future Scenarios

**FIGURE 5:**
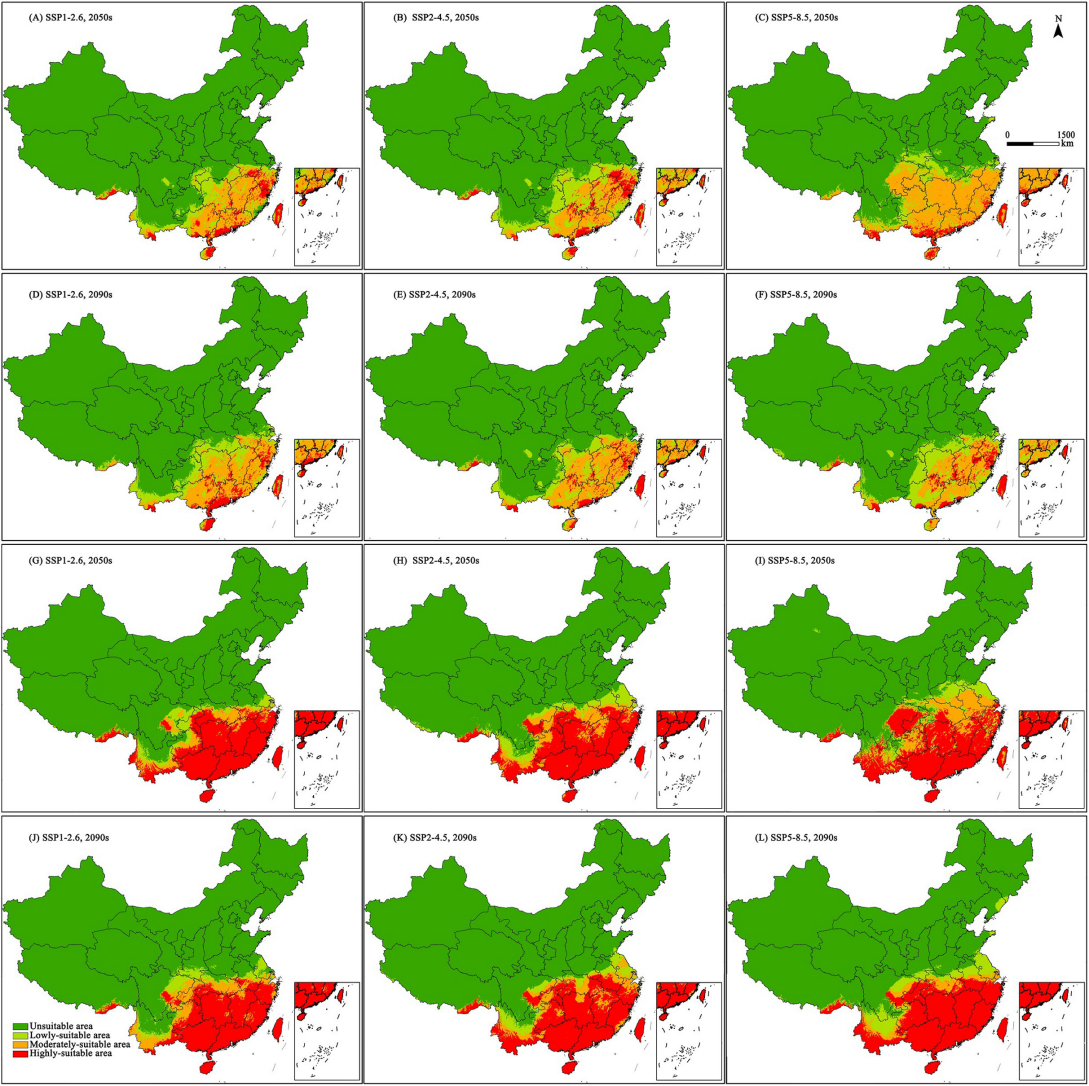
Potential distribution of *Ceracris nigricornis* in China under future scenarios using MaxEnt model (A–F) and GARP model (G–L) according to different SSP scenarios and year. SSP, shared socio‐economic pathways.

According to the results of the MaxEnt model (Figure 5A–F and Table 3), we can know that the changes of suitable area of *C. nigricornis* are different from current to the 2050s and from the 2050s to the 2090s under different climate scenarios. The suitable areas of *C. nigricornis* will decrease by 3.19% in the 2050s and then increase by 12.04% in the 2090s under the SSP1‐2.6 scenario. It will increase by 5.79% in the 2050s and then decrease by 7.53% in the 2090s under the SSP2‐4.5 scenario, whereas it will increase by 33.03% in 2050s and then decrease by 23.31% in the 2090s under SSP5‐8.5 scenario. Although the situation and time are different, but the suitable areas are still concentrated in the Yangtze‐Huaihe river basin, the lower‐middle reaches of the Yangtze River, South China and Southwest China. Similarly, from the results of the GARP model (Figure 5G–L and Table 3), we can know that the suitable areas of *C. nigricornis* will increase by 8.41% in 2050s and then continue to increase by 6.11% in 2090s under SSP1‐2.6 scenario. It will increase by 23.84% in the 2050s and then decrease by 0.88% in the 2090s under the SSP2‐4.5 scenario, whereas it will increase by 34.37% in 2050s and then decrease by 1.75% in 2090s under SSP5‐8.5 scenario. As climate scenarios and time change, the boundaries of suitable areas will expand to the north and southwest of China, the most obvious provinces are Sichuan, Chongqing and Yunnan.

**TABLE 3:** Potential suitable area of *Ceracris nigricornis* in China under different scenarios using MaxEnt model and GARP model (Unit: ×10^4^ km^2^).

Model type	Scenario, times	Unsuitable area	Lowly‐suitable area	Moderately‐suitable area	Highly‐suitable area
MaxEnt	Current	832.76	58.39	50.76	19.76
	SSP1‐2.6, 2050s	836.89	44.68	58.49	21.62
	SSP2‐4.5, 2050s	825.31	61.90	51.63	22.84
	SSP5‐8.5, 2050s	790.18	44.16	111.30	16.03
	SSP1‐2.6, 2090s	821.86	53.75	65.43	20.64
	SSP2‐4.5, 2090s	835.58	43.09	65.95	17.06
	SSP5‐8.5, 2090s	830.16	59.05	53.45	19.02
GARP	Current	795.51	19.73	28.79	117.57
	SSP1‐2.6, 2050s	781.56	26.81	28.97	124.27
	SSP2‐4.5, 2050s	755.91	32.45	40.43	132.80
	SSP5‐8.5, 2050s	738.42	35.10	54.97	133.11
	SSP1‐2.6, 2090s	770.55	29.30	41.01	120.75
	SSP2‐4.5, 2090s	757.72	27.90	40.17	135.81
	SSP5‐8.5, 2090s	742.33	43.49	26.47	149.31

For the highly‐suitable areas from MaxEnt model, it can be seen that these regions is small and scattered in southern China. Under SSP1‐2.6 scenario, its area will increase by 1.86 × 10^4^ km^2^ from current to the 2050s, whereas it will decrease by 0.98 × 10^4^ km^2^ from the 2050s to the 2090s. Under SSP2‐4.5 scenario, its area will increase by 3.08 × 10^4^ km^2^ from current to the 2050s, whereas it will decrease by 5.78 × 10^4^ km^2^ from the 2050s to the 2090s. Under SSP5‐8.5 scenario, its area will decrease by 3.73 × 10^4^ km^2^ from current to the 2050s, whereas it will increase by 2.99 × 10^4^ km^2^ from the 2050s to the 2090s. It can be seen that the future climate change will promote the expansion of the highly‐suitable area of *C. nigricornis*, but not infinite expansion. At the same time, the area of the highly‐suitable areas will have an adaptation process to the sudden warming of climate. Similarly, it can be seen from GARP model that these regions is big and concentrated in southern China. Under SSP1‐2.6 scenario, its area will increase by 6.7 × 10^4^ km^2^ from current to the 2050s, whereas it will decrease by 3.52 × 10^4^ km^2^ from the 2050s to the 2090s. Under SSP2‐4.5 scenario, its area will increase by 15.24 × 10^4^ km^2^ from current to the 2050s, whereas it will increase by 3.01 × 10^4^ km^2^ from the 2050s to the 2090s. Under SSP5‐8.5 scenario, its area will increase by 15.55 × 10^4^ km^2^ from current to the 2050s, whereas it will increase by 16.2 × 10^4^ km^2^ from the 2050s to the 2090s. From the predicted results of this model, the impact of future climate change on *C. nigricornis* is consistent with the predicted results of Maxent model, that is, the future climate change will promote the expansion of the highly‐suitable areas, and the area of the highly‐suitable areas will have an adaptation process to the sudden warming of climate.

For the moderately‐suitable areas from MaxEnt model, it can be seen that these areas are still distributed in southern China, but the area is twice more than that of the highly‐suitable areas. Under SSP1‐2.6 scenario, its area will increase by 7.73 × 10^4^ km^2^ from current to the 2050s, whereas it will increase by 6.95 × 10^4^ km^2^ from the 2050s to the 2090s. Under SSP2‐4.5 scenario, its area will increase by 0.88 × 10^4^ km^2^ from current to the 2050s, whereas it will increase by 14.31 × 10^4^ km^2^ from the 2050s to the 2090s. Under SSP5‐8.5 scenario, its area will increase by 60.54 × 10^4^ km^2^ from current to the 2050s, whereas it will decrease by 57.85 × 10^4^ km^2^ from the 2050s to the 2090s. It can be seen that the moderately‐suitable area decreased from 2050s to 2090s under SSP‐8.5 scenario, whereas the other scenarios and time periods all increased. It also means the future climate change will be more conducive to the expansion of the moderately‐suitable areas. Similarly, it can be seen from GARP model that these regions are mainly distributed in the northern part of the highly‐suitable area. Under SSP1‐2.6 scenario, its area will increase by 0.18 × 10^4^ km^2^ from current to the 2050s, whereas it will increase by 12.04 × 10^4^ km^2^ from the 2050s to the 2090s. Under SSP2‐4.5 scenario, its area will increase by 11.64 × 10^4^ km^2^ from current to the 2050s, whereas it will decrease by 0.26 × 10^4^ km^2^ from the 2050s to the 2090s. Under SSP5‐8.5 scenario, its area will increase by 26.18 × 10^4^ km^2^ from current to the 2050s, whereas it will decrease by 28.5 × 10^4^ km^2^ from the 2050s to the 2090s.

## 4 Discussion

Based on MaxEnt model and GARP model, the paper predicted the suitable areas of *C. nigricornis* in China, and analyzed the appropriate distribution degree of *C. nigricornis* in different regions. It was found that the prediction results of two models could reflect the distribution of *C. nigricornis* well, but the AUC value of MaxEnt model were higher than that of GARP model. The distribution area predicted by GARP model is wider, which can better reflect the range of potential distribution area, but it may have a high false positive rate, while the result predicted by MaxEnt model is conservative, but may be more accurate (Cui et al. 2016; Zhang et al. 2016). Therefore, combining the predictions of MaxEnt and GARP can take advantage of each other's strengths to gain a more complete picture of the potential distribution of *C. nigricornis*.

Insects are very sensitive to changes in the external environment such as temperature, precipitation or humidity (Cai, Pang, and Yang 2001), so climate change will have an important impact on the survival and development of insects. Temperature variation will affect the growth and development of insects (overwintering survival rate), metabolic rate, survival rate, reproduction rate and other life activities, whereas precipitation changes have direct and indirect effects on insects, such as heavy rainfall directly affects the population size of small insects or indirectly affects insects by changing the relative humidity of the air (Guo, Ma, and Kang 2023; Youngblood et al. 2023). According the study, the important factors affecting the distribution of *C. nigricornis* are min temperature of coldest month (bio6), mean temperature of coldest quarter (bio11), precipitation of driest month (bio14) and precipitation of wettest quarter (bio16). *C. nigricornis* overwinter with eggs, and begin to hatch in late April. This period of time coincides with the coldest season, so min temperature of coldest month and mean temperature of coldest quarter determines whether it can survive the winter and hatch safely. *C. nigricornis* is an insect that draw water from plants and do not need additional water. So as long as there is vegetation, drought will have no effect on its growth. In contrast, dry conditions are more conducive to incubation (Wu, Hao, and Kang 2021). In southern China, the dry season is usually from February to April, when locust eggs hatch. Therefore, precipitation of wettest quarter (bio14) is important factors affecting the survival of *C. nigricornis* (Chang et al. 2008). Similarly, June to August is the key period for adult emergence, and insufficient precipitation will increase the risk of water loss of larva (Dang and Chen 2011).

*Ceracris nigricornis* is a host‐oriented leaf‐eating pest, its growth, development and distribution are closely related to host species (Zhang et al. 2002). Hunan, Jiangxi, Fujian, Zhejiang, Anhui, Sichuan, Zhejiang and Guangdong are the main production areas of bamboo resources (Li and Feng 2019), this is consistent with the predicted distribution of *C. nigricornis* in this study. From the prediction results of MaxEnt model and GARP model under current scenario, the highly‐suitable areas for *C. nigricornis* are the southern part of Anhui, the western part of Zhejiang, the northeastern part of Guangxi, the eastern of Hainan and Taiwan. Therefore, it is suggested that the local forestry authorities should strengthen the monitoring of *C. nigricornis* to prevent it from causing serious harm to bamboo forests. In future climate scenarios, it can be seen that climate warming will promote the expansion of the suitable area, but not infinite expansion. The potential distribution of *C. nigricornis* in China is still in the south of Qinling‐Huaihe River, which may indicate that the low temperature in winter in the northern region is still in the range that restricts the migration of *C. nigricornis* to the north. To the new highly‐suitable or moderately‐suitable areas of province such as Sichuan, Chongqing and Yunan, should be strengthened to quarantine inspection and pest monitoring in combination with the spatial and temporal distribution of bamboo.

In this study, MaxEnt model and GARP model were used to predict the suitable habitats of *C. nigricornis* in China by combining bioclimatic variables, elevation and actual site data. However, there are still some problems. For example, the impact of soil factors on food sources for food moths has not been fully considered, and the area of non‐growing areas such as water systems and towns has not been eliminated. This may inevitably increase the niche width. Meanwhile, some parameters in this study are default, which may lead to low expressive ability of the model (Sillero et al. 2021). Therefore, we will strengthen this consideration to make the prediction of the potential distribution of species more realistic in future studies.

## Author Contributions

**Chun Fu:** data curation (equal), formal analysis (equal), investigation (equal), software (equal), validation (equal), visualization (equal), writing – original draft (lead). **Xuanye Wen:** formal analysis (equal), investigation (equal), resources (equal), validation (equal), writing – review and editing (equal). **Tingting Huang:** data curation (lead), methodology (equal), software (equal), validation (equal). **Yanli Wang:** data curation (equal), software (equal), validation (equal). **Xiu Liu:** data curation (equal), investigation (equal), software (equal). **Na Jiang:** formal analysis (equal), validation (equal). **Rulin Wang:** conceptualization (equal), project administration (equal), supervision (equal), visualization (equal), writing – review and editing (equal). **Jinpeng Zhao:** conceptualization (equal), methodology (equal), resources (equal), supervision (equal), visualization (equal), writing – review and editing (equal).

## Conflicts of Interest

The authors declare no conflicts of interest.

## Supporting information


Table S1

Table S2

## Data Availability

All data are in the main text or supporting information.

## References

[ece370439-bib-0001] Busby, J. R. 1991. BIOCLIM‐A Bioclimate Analysis and Prediction System. Nature Conservation: Cost Effective Biological Surveys and Data Analysis. Melbourne: CSIRO.

[ece370439-bib-0002] Cai, W. Z. , X. F. Pang , and D. Yang . 2001. General Entomology. Beijing: China Agricultural University Press.

[ece370439-bib-0003] Carpenter, G. , A. N. Gillison , and J. Winter . 1993. “DOMAIN: A Flexible Modelling Procedure for Mapping Potential Distributions of Plants and Animals.” Biodiversity and Conservation 2, no. 6: 667–680.

[ece370439-bib-0004] Chang, X. N. , H. J. Gao , F. J. Chen , and B. P. Zhai . 2008. “Effects of Environmental Moisture and Precipitation on Insects: A Review.” Chinese Journal of Ecology 27: 619–625.

[ece370439-bib-0005] Cui, X. Y. , W. J. Wang , X. Q. Yang , S. Li , S. Y. Qin , and R. Jun . 2016. “Potential Distribution of Wild *Camellia oleifera* Based on Ecological Niche Modeling.” Biodiversity Science 24, no. 10: 1117–1128.

[ece370439-bib-0006] Dang, Z. H. , and F. J. Chen . 2011. “Responses of Insects to Rainfall and Drought.” Chinese Journal of Applied Entomology 48, no. 5: 1161–1169.

[ece370439-bib-0007] Etherington, T. R. 2011. “Python Based GIS Tools for Landscape Genetics: Visualising Genetic Relatedness and Measuring Landscape Connectivity.” Methods in Ecology and Evolution 2, no. 1: 52–55.

[ece370439-bib-0008] Feng, J. D. , M. Wang , D. Feng , Z. W. Feng , Y. F. Wen , and P. Chen . 2024. “Comparison of Morphological and Biological Characteristics of Two Bamboo Insects Adults in the Same Area.” Forest Research 37, no. 2: 81–89.

[ece370439-bib-0009] Gao, S. 2017. Phylogenetic Analysis and Molecular Ecology of Bamboothracris. Nanjing: Nanjing Normal University.

[ece370439-bib-0010] Gao, S. , G. F. Jiang , and B. B. Fan . 2020. “Gene Introgreesion Between Two Species of Sympatric Bamboo Locusts Based on Microsatellite Markers.” Journal of Economic Entomology 42, no. 3: 566–572.

[ece370439-bib-0011] GBIF . 2023. “*Ceracris nigricornis* Walker, 1870 in GBIF Secretariat.” Checklist dataset. 10.15468/39omei, GBIF.org.

[ece370439-bib-0012] Guo, W. , C. Ma , and L. Kang . 2023. “Community Change and Population Outbreak of Grasshoppers Driven by Climate Change.” Current Opinion in Insect Science 61: 101154. 38104960 10.1016/j.cois.2023.101154

[ece370439-bib-0013] Guo, Y. X. , Y. F. Wang , Z. X. Fu , and X. Ma . 2022. “Prediction and Analysis of Potential Geographical Distribution of *Bunias orientalis* in China Based on the Optimized MaxEnt Model.” Plant Protection 48, no. 2: 40–47.

[ece370439-bib-0014] Haase, C. G. , A. Yang , K. M. McNyset , and J. K. Blackburn . 2021. “GARPTools: R Software for Data Preparation and Model Evaluation of GARP Models.” Ecography 44: 1790–1796.

[ece370439-bib-0015] He, Q. J. 2002. Study on Biological Characteristics of Ceracris nigricornis Walker and Ceracris fasciata (Br. ‐ W.). Kunming: Southwest Forestry College.

[ece370439-bib-0016] Hirzel, A. H. , J. Hausser , D. Chessel , and N. Perrin . 2002. “Ecological Niche Factor Analysis: How to Compute Habitat‐Suitability Maps Without Absence Data.” Ecology 83, no. 7: 2027–2036.

[ece370439-bib-0017] Jiménez‐Valverde, A. , and J. M. Lobo . 2007. “Threshold Criteria for Conversion of Probability of Species Presence to Either‐Or Presence‐Absence.” Acta Oecologica 31, no. 3: 361–369.

[ece370439-bib-0018] Jin, C. X. , C. Jiang , and X. Y. Zhang . 2022. “Evaluation and Projection of Temperature in Southwestern China by CMIP6 Models.” Chinese Journal of Agrometeorology 43, no. 8: 597–611.

[ece370439-bib-0019] Li, Y. M. , and P. F. Feng . 2019. “Bamboo Resources in China Based on the Ninth National Forest Inventory Data.” World Bamboo and Rattan 17, no. 6: 45–48.

[ece370439-bib-0020] Liu, X. Y. , D. X. Zhang , and X. G. He . 2024. “Unveiling the Role of Climate in Spatially Synchronized Locust Outbreak Risks.” Science Advances 10, no. 7: eadj1164. 38354233 10.1126/sciadv.adj1164 PMC10866544

[ece370439-bib-0021] Lobo, J. M. , A. Jiménez‐Valverde , and R. Real . 2008. “AUC: A Misleading Measure of the Performance of Predictive Distribution Models.” Global Ecology and Biogeography 17: 145–151.

[ece370439-bib-0022] Merow, C. , M. J. Smith , and J. A. Silander . 2013. “A Practical Guide to MaxEnt for Modeling Species Distributions: What It Does, and Why Inputs and Settings Matter.” Ecography 36, no. 10: 1058–1069.

[ece370439-bib-0023] Phillips, S. J. , R. P. Anderson , and R. E. Schapire . 2006. “Maximum Entropy Modeling of Species Geographic Distributions.” Ecological Modelling 190: 231–259.

[ece370439-bib-0024] Phillips, S. J. , and M. Dudik . 2008. “Modeling of Species Distributions With MaxEnt: New Extensions and a Comprehensive Evaluation.” Ecography 31: 161–175.

[ece370439-bib-0025] Sillero, N. , S. Arenas‐Castro , U. Enriquez‐Urzelai , et al. 2021. “Want to Model a Species Niche? A Step‐By‐Step Guideline on Correlative Ecological Niche Modelling.” Ecological Modelling 456: 109671.

[ece370439-bib-0026] Sillero, N. , and A. M. Barbosa . 2021. “Common Mistakes in Ecological Niche Models.” International Journal of Geographical Information Science 35, no. 2: 213–226.

[ece370439-bib-0027] Stockwell, D. , and D. Peters . 1999. “The GARP Modelling System: Problems and Solutions to Automated Spatial Prediction.” International Journal of Geographical Information Science 13: 143–158.

[ece370439-bib-0028] Sun, Y. , D. H. Qin , and H. B. Liu . 2012. “Introduction to Treatment of Uncertainties for IPCC Fifth Assessment Report.” Climate Change Research 8, no. 2: 150–153.

[ece370439-bib-0029] Wang, Q. Q. 2019. Geometric Morphometrics and Subspecific Differentiation of Bamboothracris. Shaanxi: Shaanxi Normal University.

[ece370439-bib-0030] Wang, Y. G. , H. B. Zhu , and W. C. Xu . 2021. “A Review on ROC Curve and Analysis.” Journal of Guangdong University of Technology 38, no. 1: 46–53.

[ece370439-bib-0031] Wang, Y. S. , B. Y. Xie , F. H. Wan , Q. M. Xiao , and L. Y. Dai . 2007. “Application of ROC Curve Analysis in Evaluating the Performance of Alien Species' Potential Distribution Models.” Biodiversity Science 15, no. 4: 365–372.

[ece370439-bib-0032] Wu, J. Q. 2005. “Egg‐Laying Habit and Survey Method for Ovum Dollop of *Ceracris nigricornis* .” Entomological Journal of East China 14: 311–314.

[ece370439-bib-0033] Wu, T. J. , S. G. Hao , and L. Kang . 2021. “Effects of Soil Temperature and Moisture on the Development and Survival of Grasshopper Eggs in Inner Mongolian Grasslands.” Frontiers in Ecology and Evolution 9: 727911.

[ece370439-bib-0034] Xu, J. Y. 2024. “On the Bias in the AUC Variance Estimate.” Pattern Recognition Letters 178: 62–68. 38186922 10.1016/j.patrec.2023.12.012 PMC10768968

[ece370439-bib-0035] Xu, Z. L. , H. H. Peng , and S. Z. Peng . 2015. “The Development and Evaluation of Species Distribution Models.” Acta Ecologica Sinica 35, no. 2: 557–567.

[ece370439-bib-0036] Youngblood, J. P. , A. J. Cease , S. Talal , et al. 2023. “Climate Change Expected to Improve Digestive Rate and Trigger Range Expansion in Outbreaking Locusts.” Ecological Monographs 93, no. 1: e1550.

[ece370439-bib-0037] Yuan, H. , H. H. Chang , L. N. Zhao , C. Yang , and Y. Huang . 2019. “Sex‐ and Tissue‐Specific Transcriptome Analyses and Expression Profiling of Olfactory‐Related Genes in *Ceracris nigricornis* Walker (Orthoptera: Acrididae).” BMC Genomics 20, no. 1: 808. 31694535 10.1186/s12864-019-6208-x PMC6836668

[ece370439-bib-0038] Zhang, C. , L. Chen , C. M. Tian , T. Li , R. Wang , and Q. Q. Yang . 2016. “Predicting the Distribution of Dwarf Mistletoe (*Arceuthobium sichuanense*) With GARP and MaxEnt Models.” Journal of Beijing Forestry University 38, no. 5: 23–32.

[ece370439-bib-0039] Zhang, F. P. , Q. L. Chen , S. L. Chen , Y. M. Hou , and M. S. You . 2002. “Research Advances on the Pests That Eat Leaves of *Phyllostachys heterocycla cv. pubescens* .” Journal of Bamboo Research 21, no. 3: 55–60.

[ece370439-bib-0040] Zhou, T. J. , L. W. Zou , and X. L. Chen . 2019. “Commentary on the Coupled Model Intercomparison Project Phase 6 (CMIP6).” Climate Change Research 15, no. 5: 445–456.

[ece370439-bib-0041] Zhu, G. P. , Q. Liu , and Y. B. Gao . 2014. “Improving Ecological Niche Model Transfer Ability to Predict the Potential Distribution of Invasive Exotic Species.” Biodiversity Science 22, no. 2: 8.

